# Gelatinase Biosensor Reports Cellular Remodeling During Epileptogenesis

**DOI:** 10.3389/fnsyn.2020.00015

**Published:** 2020-04-21

**Authors:** Nathalie Bouquier, Benoit Girard, Juri Aparicio Arias, Laurent Fagni, Federica Bertaso, Julie Perroy

**Affiliations:** IGF, Université de Montpellier, CNRS, INSERM, Montpellier, France

**Keywords:** activatable cell-penetrating peptides (ACPPs), gelatinase, matrix metalloproteinases (MMPs), epileptogenesis, kainate (KA), molecular imaging probes

## Abstract

Epileptogenesis is the gradual process responsible for converting a healthy brain into an epileptic brain. This process can be triggered by a wide range of factors, including brain injury or tumors, infections, and status epilepticus. Epileptogenesis results in aberrant synaptic plasticity, neuroinflammation and seizure-induced cell death. As Matrix Metalloproteinases (MMPs) play a crucial role in cellular plasticity by remodeling the extracellular matrix (ECM), gelatinases (MMP-2 and MMP-9) were recently highlighted as key players in epileptogenesis. In this work, we engineered a biosensor to report *in situ* gelatinase activity in a model of epileptogenesis. This biosensor encompasses a gelatinase-sensitive activatable cell penetrating peptide (ACPP) coupled to a TAMRA fluorophore, allowing fluorescence uptake in cells displaying endogenous gelatinase activities. In a preclinical mouse model of temporal lobe epilepsy (TLE), the intrahippocampal kainate injection, ACPPs revealed a localized distribution of gelatinase activities, refining temporal cellular changes during epileptogenesis. The activity was found particularly but not only in the ipsilateral hippocampus, starting from the CA1 area and spreading to dentate gyrus from the early stages throughout chronic epilepsy, notably in neurons and microglial cells. Thus, our work shows that ACPPs are suitable molecular imaging probes for detecting the spatiotemporal pattern of gelatinase activity during epileptogenesis, suggesting their possible use as vectors to target cellular reactive changes with treatment for epileptogenesis.

## Introduction

The biology of excitatory synapses relies on its tetrapartite organization, which includes pre- and post-synaptic neurons, glial cells and the extracellular matrix (ECM) stabilizing synaptic contacts. Matrix Metalloproteinases (MMPs) are key components of the ECM and constitute a large family of proteases, most of which act in the extracellular space (Lukasiuk et al., 2011). Among them, MMP-2 and MMP-9 constitute the class of gelatinases and are amongst the most abundant MMPs in the brain. They have emerged as regulators of diverse biological processes under normal and pathological conditions. These secreted endopeptidases have a significant role in extracellular proteolytic processes at the excitatory synapses. In particular, MMP-9 contributes to the regulation of structural and functional synaptic plasticity (Nagy et al., 2006; Szepesi et al., 2013). It is locally secreted at the level of dendritic spines in an activity-dependent manner (Konopacki et al., 2007), where it cleaves synaptic cell adhesion molecules and cell surface receptors (Tian et al., 2007; Szepesi et al., 2014), thus shaping dendritic spine morphology (Michaluk et al., 2009, 2011). Hence, disruption of the ECM allows structural and functional synaptic plasticity under physiological conditions, but can also, after an initial brain damage, induce aberrant molecular and cellular remodeling. These molecular modifications, which are still poorly understood, trigger cellular disturbances and disorganization of the networks that can lead to an epileptic brain over weeks.

Epilepsy is a brain disorder characterized by spontaneous and recurrent seizures due to hypersynchronous and excessive neuronal activities. In particular, temporal lobe epilepsy (TLE), the most widespread form of focal epilepsy, arises from epileptic foci located in the temporal neocortex and hippocampus. So far, therapeutic efforts were focused on treating symptoms, such as seizures. However, 30% of TLE patients are still resistant to currently available anti-epileptic drugs, a percentage that has not changed in decades despite the new medication. Hence, new treatments rather targeting the underlying disease mechanisms of the epilepsy pathogenesis should be explored (Terrone et al., 2016; Łukawski et al., 2018). This strategy goes through a better understanding of the establishment of the disease. Epileptogenesis is the mechanism leading to chronic seizures (Pitkänen and Engel, 2014). This dynamic process takes place after an initial insult and converts healthy brain tissue into an epileptic one, with hyper synchronization and hyperexcitability of the neuronal network. It involves a cascade of biological events such as neuronal cell death, proliferation, neuroinflammation, disruption of the blood-brain barrier, neuronal network reorganization and aberrant synaptic plasticity (Pitkänen and Sutula, 2002; Gorter et al., 2015; Łukawski et al., 2018). All these cellular reactive changes, which contribute to the formation of ictogenic neuronal networks, are hereafter included in the term “remodeling.” The ECM is a structural scaffold that plays an important role in this detrimental rearrangement (Pitkänen et al., 2014).

Cumulative evidence indicates that gelatinases, and particularly MMP-9, play a fundamental function in epileptogenesis (Ikonomidou, 2014; Khomiak and Kaczmarek, 2018). MMP-9 is upregulated in several epilepsy animal models (Wilczynski et al., 2008) as well as in epileptic patients (Quirico-Santos et al., 2013; Acar et al., 2015). Intrahippocampal injection of kainate (KA) in rodents is an isomorphic epileptogenesis model, which mimics and recapitulates the main clinical features of human TLE. It induces a prolonged seizure, named status epilepticus, that triggers the epileptogenic process, leading to chronic seizures. Evidence of gelatinase implication in seizures was first described by Zhang et al. (1998) showing their upregulation after KA stimulation of rat brain, especially in the hippocampus. The increase of MMP-9 expression (at the mRNA and protein levels) and elevated enzymatic activity in the dentate gyrus play a role in epileptic focus formation (Szklarczyk et al., 2002; Jourquin et al., 2003). MMP-9 was thus proposed as a biomarker to investigate epileptogenesis (Yin et al., 2011; Bronisz and Kurkowska-Jastrzębska, 2016). Because gelatinases are released in a specific time and space-dependent manner at excitatory synapses, precise information about their kinetics of activation is necessary to propose new therapeutic strategies.

In this study, we examined gelatinase activities throughout the epileptogenesis process using an approach based on activatable cell-penetrating peptides (ACPPs). Gelatinase-based ACPPs have already been handled for imaging tumors (Olson et al., 2009, 2010; Nguyen et al., 2010) and ischemic stroke (Chen et al., 2017). We used them in an *in vivo* model of KA-induced epileptogenesis to delineate the gelatinase spatiotemporal activation profile. Not only this tool is of particular interest to finely localize cellular reactive changes during epileptogenesis, but it could also open opportunity for selective and local delivery of therapeutic agents targeted by gelatinase activity.

## Materials and Methods

### Peptide Synthesis

Two peptides were designed from the original publication by Jiang et al. (2004). MMP-2/-9 cleavable ACPP presents the following amino acid sequence: Suc-e8-(Ahx)-PLGLAG-r9-(Ahx)-k(TAMRA)-NH2. As a negative control, a cleavable-resistant ACPP with scrambled linker was synthesized: Suc-e8-(Ahx)-LALGPG-r9-k(Cy5)-NH2. Ahx is a 6-aminohexanoic acid, a flexible hydrophilic linker to facilitate hairpin conformation. Capital letters indicate L-form amino acids and lowercase letters, D-form amino acids. Peptides were N-terminally capped with a succinyl (Suc) group to provide a ninth negative charge equivalent to glutamate without an amino group, and C-termini were amidated. The C-termini were labeled with TAMRA fluorophore coupled to a D-lysine k (Smart Bioscience, Saint-Egrève, France). Peptides were synthesized on a Symphony Synthesizer (Protein Technologies Inc., Tucson, AZ, USA), at a 0.1 mmol scale on a CTC resin (substitution approx. 1.6 mmol/g) and using TAMRA labeled Lysine. Fmoc protecting group was removed using 20% piperidine in DMF and free amine was coupled using ten fold excess of Fmoc amino acids and HCTU/DIEA activation in NMP/DMF (3 × 15 min). The peptide was deprotected and cleaved from the resin with TFA/H2O/1,3-dimethoxybenzene/TIS 92.5/2.5/2.5/2.5 (vol.), then precipitated out in cold diethyl ether. The resulting white solids were washed two times with diethyl ether, resuspended in H_2_O/acetonitrile and freeze-dried to afford crude peptide. Finally, fluorophore-labeled peptides were purified by HPLC (C18 reverse-phase column, eluted with 10–40% acetonitrile in water with 0.1% CF3COOH) and lyophilized overnight. The molecular weight of all peptides was confirmed by mass spectroscopy (LC-ESI-MS), and the concentration of each peptide stock solution was verified by UV-vis absorbance.

### Cell Culture

Primary cultures of hippocampal neurons were prepared from E18 Wistar rat embryos (Janvier Labs). Briefly, hippocampi were dissected, treated with 0, 05% trypsin-EDTA, and mechanically disrupted by 10 cycles of aspiration and ejection through a micropipette tip. Dissociated hippocampal cells were seeded on coverslips in 35 mm dishes precoated with 50 μg/ml poly-D-lysine (Sigma–Adrich), in Neurobasal medium containing 2% B27 supplement, 10% heat-inactivated horse serum, 0.5 mM glutamine, and antibiotics (100 U/ml penicillin and 100 mg/ml streptomycin; Gibco). Neurons were maintained in water-saturated 95% air/5% CO_2_ at 37°C. The seeding medium was replaced after 20 h with a serum-free neuronal culture medium. After 10 days of culture, the neurons were enriched by treatment with 5 μM cytosine b-D-arabinofuranoside hydrochloride (Sigma–Adrich) for 72 h. The cultures were used for experiments 15 days after plating.

### Activation of Gelatinases in Cultures of Hippocampal Neurons

Activation of gelatinases in cultured neurons was performed by exposure to NMDA or glutamate: cells were washed three times with EBSS containing Ca^2+^, and then stimulated with 100 μM NMDA or 50 μM glutamate for 10 min at 37°C in either absence or presence of Calcium Diethylene Triamine Penta Acetate (Ca-DTPA, 5 mM) a metal chelator and broad-spectrum MMP inhibitor. For β-Dystroglycan expression analysis, cells were further incubated for 10 or 30 min then lysed in 4X SDS sample buffer and denaturated by heating for 5 min at 95°C. For imaging of ACPPs uptake, following the transient NMDA or glutamate application, cells were incubated for 2 h 30 min with 1 μM of ACPPs and then fixed for 15 min with 4% paraformaldehyde (PFA) + 4% sucrose + Hoechst 33258 for nuclei staining. Coverslips were mounted with Mowiol for observation under an epifluorescent microscope equipped for optical sectioning (Apotome, Zeiss). The number of TAMRA stained cells was assessed with Cell Profiler, an automated image analysis software. The total number of cells was counted with blue stained nuclei (size range 6.5 μm–26 μm) as well as the number of positive red cells (size range 16 μm–40 μm) with an identical threshold of fluorescence intensity on three independent experiments (number of counted cells >2,000 per condition per experiment on a minimum of 10 fields acquired with a 10× objective).

### Western Blot

Neuronal culture lysates containing an equal total amount of protein samples were loaded on 12% SDS-polyacrylamide gels and transferred onto nitrocellulose membranes (GE Healthcare) at 40 V overnight at 4°C. After incubation for 1 h in blocking buffer (PBS, 0.1% Tween-20, and 5% dried non-fat milk), membranes were incubated for 2 h at room temperature with an anti-β-dystroglycan primary antibody (NCL-b-DG, 1:500, Novocastra). The membranes were then incubated with horseradish peroxidase-labeled secondary antibody (Jackson Immuno Research Laboratories) diluted 1:5,000 for 1 h at room temperature. After washing, the immunoblot signals were visualized by enhanced chemiluminescence detection (Western Lightning ECL-Plus, PerkinElmer) and acquired on a ChemiDoc Touch Imaging System (Bio-Rad) controlled by Image Lab software version 3.0 (Bio-Rad). After incubation in a stripping solution (PBS, 0.1% Tween-20, 0.5% sodium azide), the same membranes were re-blotted with an anti-GAPDH (Sigma–Adrich, St. Louis, MO, USA, 1:30,000, G9545) for loading control. For quantification of changes in protein expression levels, band intensities were measured with ImageJ software. The optical density values of β-dystroglycan were normalized to those of GAPDH bands in the same sample and expressed as a percentage of control treatment.

### Animals

A total of 58 mice were used in this study. All animal procedures were carried out following the European Communities Council Directive, approved by the French Ministry for Agriculture (2010/63/EU, file# 2017011617122099) and supervised by the IGF institute’s local Animal Welfare Unit (CEEA-LR36). Male C57BL/6 were purchased from Janvier Labs. Animals were housed under standardized conditions with a 12 h light/dark cycle, stable temperature (22 ± 2°C), controlled humidity (55 ± 10%), and food and water *ad libitum*.

### Intrahippocampal Kainate Injection Model

The stereotaxic intrahippocampal KA injection is a mouse model of mesial TLE (Bouilleret et al., 1999). Briefly, wild-type C57BL6/J adult male mice (8–16 weeks-old) were intraperitoneally anesthetized with PBS-buffered solution containing 400 mg/kg chloral hydrate, plus a local subcutaneous injection of lidocaine (Xylocaine, AstraZeneca; 4 mg/kg in 50 μl of sterile 0.9% NaCl solution) and placed in a stereotaxic frame using the David Kopf mouse adaptor. All stereotaxic injections were performed using a 10 μl micro-syringe with a stainless steel 33G beveled needle controlled by a micro-pump. Mice were injected in the right dorsal hippocampus (AP = −2; ML = −1.5; DV = −2 mm from Bregma^1^) with 50 nl of a 20 mM solution of kainic acid (KA, 1 nmol; Sigma–Adrich) in 0.9% sterile NaCl. After recovery, the animals were kept under observation for 8–10 h post-injection and displayed non-convulsive status epilepticus defined by characteristic behavioral pattern (long-lasting period of immobility, head deviations, and asymmetric forelimbs movements or rotations). All the KA-injected mice showed DG dispersion when analyzed post-mortem. Control animals were injected with 50 nl of 0.9% sterile NaCl (saline solution) under the same conditions. Stereotaxic injections of ACPP peptide (0.2 nmol, 1 μl at 200 μM) at 200 nl/min were performed ipsilateral and contralateral of saline solution or KA injection (AP = −2.5; ML = ±1.5; DV = −2 mm from Bregma) 7 days before sacrifice. For all ACPPs injection experiments, a total of 52 mice were used. Three independent experiments were performed, with 2–3 animals injected per condition for each experiment.

For EEG recording, six mice were implanted right after KA intrahippocampal injection, at the same coordinates with a bipolar tungsten electrode, as well as skull cortical electrodes on the frontoparietal bone and a reference electrode on the occipital bone. EEG activity was recorded as previously described (Girard et al., 2019) every 2 days during the first week, and then once a week. Briefly, micro connectors on freely moving animals were plugged into an EEG amplifier (Pinnacle Technology Inc.). EEG recordings were performed with parallel video monitoring of animal behavior. Hippocampal seizures were automatically detected using pClamp^®^software. The threshold for detection of paroxystic activities was set at 3-fold the standard deviation of the signal amplitude without seizures and a 10 ms event duration. Peak detection was visually checked a posteriori. Traces of EEG recordings were classified according to severity scores: score 0: normal activity (control state); score 1: low-voltage background activity; score 2: spikes; score 3: short discharges; score 4: long discharges; score 5: recurrent seizures; score 6: secondarily generalized seizures. Example traces in Figure 3 illustrate the progression of seizures from KA injection up to the chronic phase.

**Figure 1:**
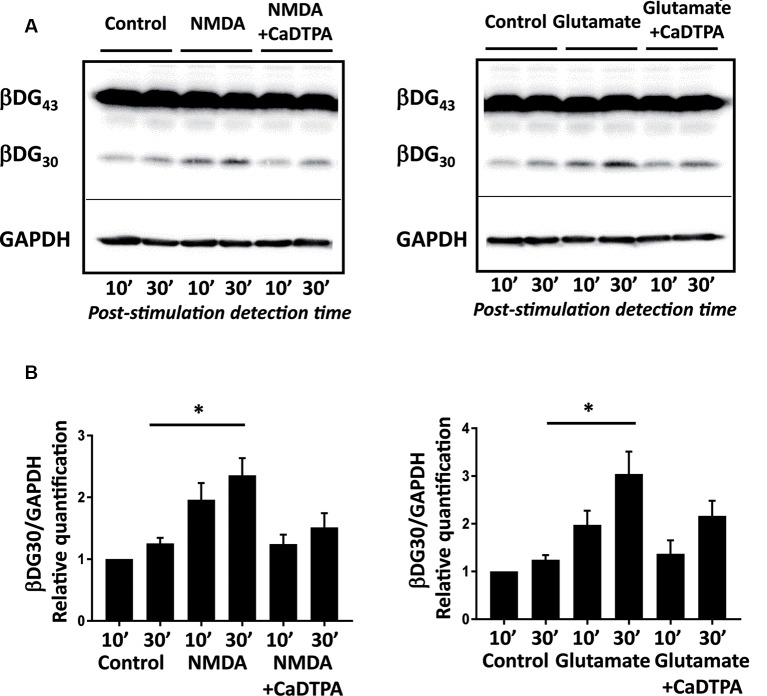
NMDA or glutamate stimulations activate endogenous gelatinases in cultured neurons. (**A)** Rat hippocampal neurons (14 DIV) were stimulated for 10 min with NMDA 100 μM or glutamate 50 μM with or without Ca-DTPA, a metal chelator and a broad range inhibitor of MMPs activity, then incubated for 10 or 30 min before lysis. Endogenous gelatinase activation was detected by cleavage of β-dystroglycan (β-DG), an MMP-9 substrate, by Western Blot. **(B)** Quantification of 30 kD β-DG cleavage product relative to GAPDH as a protein loading control. Graph represents mean values ± SEM (*n* = 4 independent experiments). Non-parametric Kruskal–Wallis test (*p*-value = 0.0018 for NMDA experiments and *p*-value = 0.0003 for glutamate experiments) followed by Dunn’s multiple comparison test where *p < 0.05 (control 30 min vs. stimulation 30 min).

**Figure 2:**
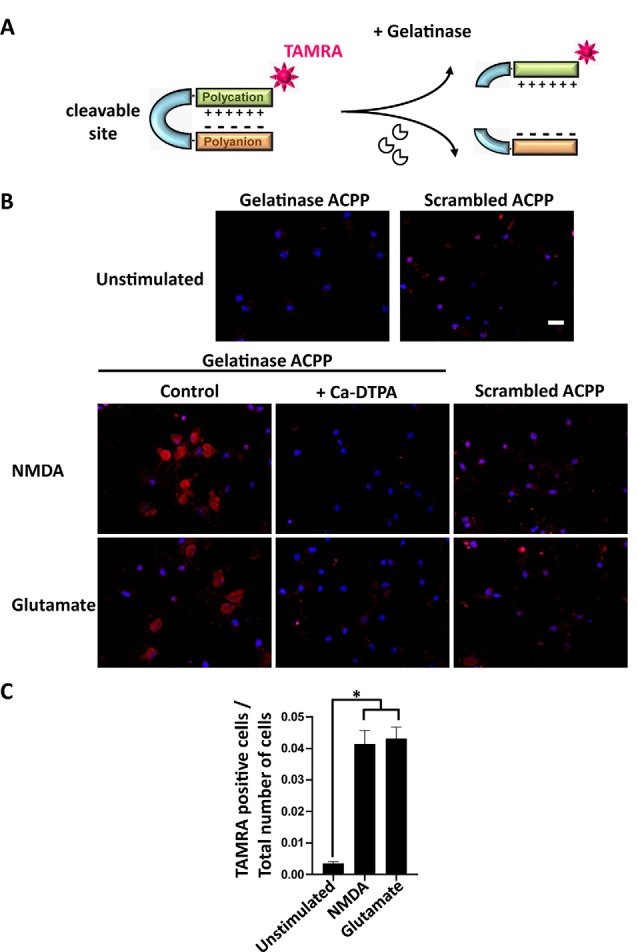
Activatable cell penetrating peptides (ACPPs) can report gelatinase activation by cellular uptake of fluorescence.** (A)** Structure of ACPPs: a cell-penetrating polycationic part (green) is attached to a polyanionic sequence (orange) *via* a cleavable linker (blue). In the presence of active gelatinases (MMP-2/-9), the ACPP is cleaved. The cell-penetrating polycationic part coupled to a TAMRA fluorophore is released and can then enter into surrounding cells. **(B)** Cellular uptake of TAMRA fluorophore induced by NMDA or glutamate transient perfusion. Rat hippocampal neurons (DIV14) were stimulated for 10 min with NMDA (100 μM) or glutamate (50 μM), with or without the broad-spectrum Matrix Metalloproteinase (MMP) inhibitor Ca-DTPA (5 mM), then incubated for 2 h 30 in presence of 1 μM gelatinase ACPPs or an ACCP containing an uncleavable scrambled sequence as a linker (scrambled-ACPP). Hoechst labeled nuclei (blue). Scale bar 20 μm. **(C)** Quantification of the number of TAMRA fluorescent cells in gelatinase-ACPP treated cultures in control, NMDA or glutamate stimulated conditions. The graph represents mean values ± SEM (*n* > 30 fields for each condition, in three independent experiments). One-way ANOVA test (*p*-value = 0.0264) was performed followed by Tukey’s multiple comparison test where *p < 0.05 (control vs. stimulations).

**Figure 3:**
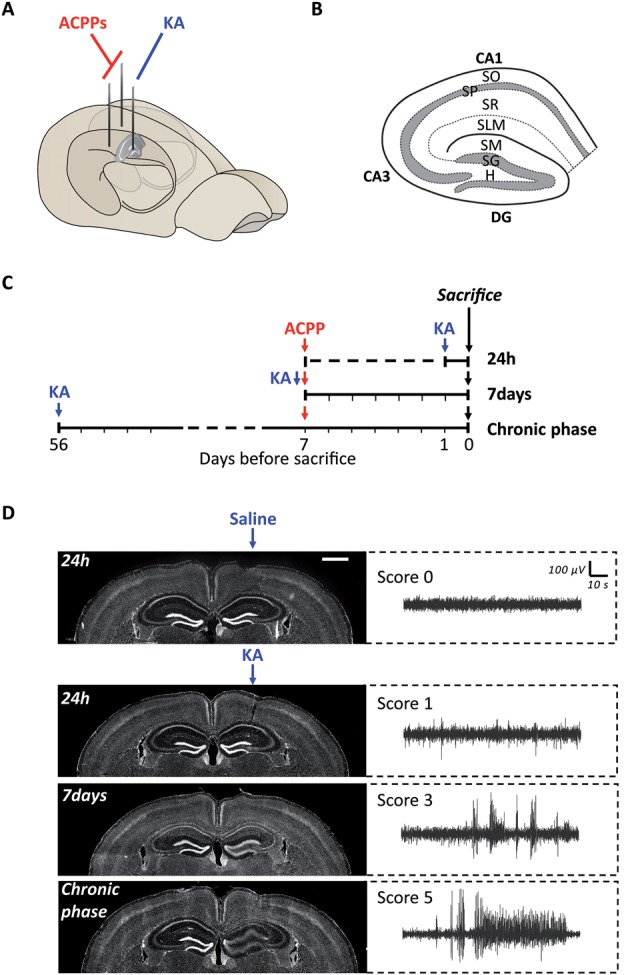
Experimental protocol of ACPPs injection in a mouse model of epileptogenesis. **(A)** Scheme of kainic acid (KA) and ACPPs injections in hippocampal hemispheres. **(B)** Scheme of hippocampal structure and lamination. CA, cornu ammonis; DG, dentate gyrus; H, hilus; SO, stratum oriens; SP, stratum pyramidale; SR, stratum radiatum; SLM, stratum lacunosum-moleculare; SM, stratum moleculare; SG, stratum granulosum. **(C)** Experimental procedure: timeline of injections. **(D)** Hoechst labeled nuclei showing progressive dentate gyrus cell dispersion in KA injected hippocampus, characteristic of epileptogenesis (scale bar: 1 mm). Corresponding EEG recordings representative of the different severity scores are shown on the right. Score 0: normal activity; score 1: low-voltage background activity; score 3: short discharges; score 5: recurrent seizures.

### Tissue Preparation and Immunohistochemistry

One day to 8 weeks after KA injection, mice were anesthetized using pentobarbital (Euthasol^®^, 400 mg/ml, injected 360 mg/kg) and transcardially perfused with 4% (w/v) PFA. Mouse brains were dissected, postfixed in PFA for 36 h and cut into serial 35 μm-thick coronal sections with a vibratome (VT1000S; Leica). Free-floating sections were rinsed three times in PBS and incubated for 20 min in PBS/0.2% Triton X-100 for permeabilization, and blocked in PBS/3% BSA for 1 h. Slices were then incubated in PBS/1% BSA/ 0.1% Triton X-100 overnight at 4°C with different primary antibodies: chicken anti-GFAP (1:300 dilution, ab4674; Abcam), rabbit anti-Iba1 (1:1,000, #019-19741; Wako), mouse anti-NeuN (1:300, MAB377; Millipore) and rabbit anti-laminin (1:300, L9393; Sigma–Adrich). The day after, sections were rinsed three times for 10 min in PBS and incubated for 2 h with fluorophore-conjugated secondary antibodies: AMCA anti-chicken 1:300, Alexa Fluor 680 anti-rabbit 1:1,000, Alexa Fluor 488 anti-mouse 1:1,000 and nuclear DNA dye Hoechst 33258. The sections were rinsed in PBS three times 10 min before mounting in DPX. At least three slices per animal were processed. Images were acquired with an AxioImager Z1 Zeiss microscope equipped for optical sectioning (Apotome) and with appropriate epifluorescence filters. All parameters were held constant for all sections and for each staining to allow comparison between samples.

### Statistical Analysis

Data are presented as the means ± SEM of at least three independent experiments. GraphPad Prism v7.02 software was used to perform statistical analyses. We used the non-parametric Kruskal–Wallis test followed by Dunn’s multiple comparison test for Western blot analysis and one-way ANOVA test followed by Tukey’s multiple comparison test for immunofluorescence quantification. Statistical significance was determined as *p < 0.05; **p < 0.01 and ***p < 0.001.

## Results

### Neuronal Excitation Induces Endogenous Gelatinase Activation

To characterize the uptake of ACPPs in neuronal cells, we first searched for suitable gelatinase activation conditions *in vitro* in primary cultures of hippocampal neurons. β-dystroglycan (β-DG) was identified previously as a native substrate of MMP-9 that is cleaved in response to enhanced neuronal activity (Michaluk et al., 2007). The proteolytic cleavage of the 43 kDa full-length transmembrane β-DG protein leads to the formation of a 30 kDa product, readily detectable by Western blot. Hence, endogenous gelatinase activation can be indirectly measured by β-DG cleavage assay. We adopted two different protocols of gelatinase activation using transient NMDA (100 μM; Tian et al., 2007), or glutamate (50 μM; Dziembowska et al., 2012) treatments for 10 min. Cleavage of β-DG increased 10 min after the end of stimulation and even more significantly 30 min later (Figure 1A; Supplementary Figures S1, S2). This cleavage was abolished by applying Ca-DTPA, a broad-spectrum MMP inhibitor (Figure 1B). Thus, NMDA or glutamate application causes the activation of endogenous gelatinases.

### ACPPs Report Endogenous Gelatinase Activation

We investigated if ACPPs could detect NMDA- or glutamate-induced endogenous gelatinase activation in neurons. ACPPs are peptidic biosensors composed of a polycationic cell-penetrating part connected to a neutralizing polyanion *via* a cleavable linker. In this hairpin conformation, the masking of positive charges prevents biosensor internalization (Figure 2A). The linker is a specific target of gelatinases. Upon gelatinases activation, proteolysis of the linker allows the dissociation of the two domains and enables the polycationic CPP to enter cells (Jiang et al., 2004; Aguilera et al., 2009). A red TAMRA fluorophore is coupled to the CPP part (Figure 2A). Hence, fluorescence uptake by the cell reports gelatinase activity.

After transient stimulation for 10 min with NMDA or glutamate, neurons were incubated with ACPPs. NMDA or glutamate stimulation strongly increased the number of TAMRA fluorescent cells compared to unstimulated neurons (Figure 2B). Ca-DTPA prevented NMDA- or glutamate-induced fluorescence uptake suggesting that gelatinase activation was involved in the process. Moreover, NMDA or glutamate stimulation failed to induce the uptake of scrambled-ACPP that cannot be cleaved by gelatinases (Figure 2B). We further quantified a 12-fold increase of gelatinase ACPPs uptake by NMDA or glutamate stimulation, compared to unstimulated condition (Figure 2C).

The coherence between results obtained by Western Blot (Figure 1) and with gelatinase biosensor in living cells (Figure 2) upon enhanced neuronal activity validates the use of ACPPs to report endogenous gelatinase activities by *in situ* detection of TAMRA fluorescence.

### ACPPs Report *in vivo* Gelatinase Activity in Epileptogenic Mouse Brain

We next used ACPPs to report gelatinase activity *in vivo*, in a mice model of epileptogenesis. We and others have previously shown that a single intrahippocampal injection of kainic acid (KA) in the right hemisphere of the brain initiates cellular remodeling leading to focal TLE (Bouilleret et al., 1999; Girard et al., 2019). We injected mice unilaterally with KA and ACPPs in both ipsilateral and contralateral sides (Figure 3A). Animals were sacrificed at three different time points after KA injection: at 24 h, after 7 days (early phase) or once seizures were spontaneous and chronic (8 weeks, chronic phase, Figure 3C). As expected, we observed the characteristic neuronal loss in the CA1 and CA3 areas of the hippocampus and the progressive granular cell dispersion in the dentate gyrus (Figure 3D). EEG recordings reported continuous progression of electrical brain activity, from low-voltage background activity 24 h after KA injection, followed by short discharges at 7 days, until recurrent, mature seizures at 8 weeks which are characteristic of an excitation/inhibition imbalance, neuronal hyperexcitability and hyper synchronization (Figure 3D).

**Figure 4:**
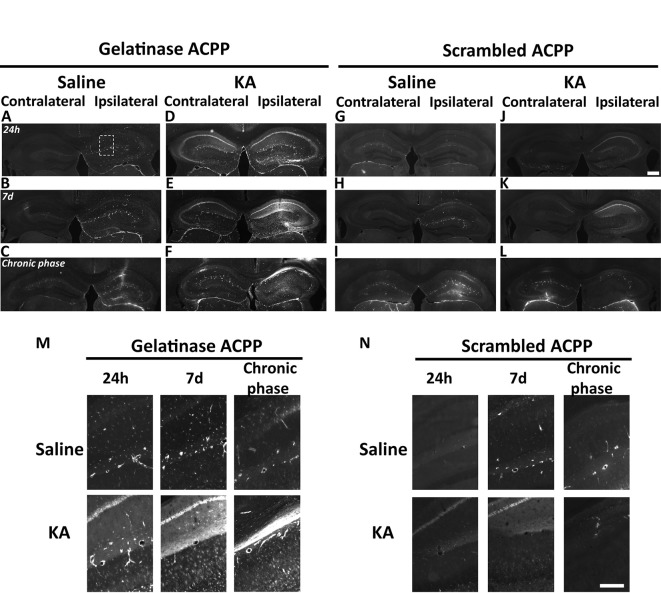
Detection of *in vivo* gelatinase activity by cleavage of ACPPs in the epileptogenic mouse brain. **(A–L)** TAMRA ACPP fluorescence in mice brains injected with KA **(D–F,J–L)** or saline solution **(A–C,G-I)** in the right hippocampus, and gelatinase-ACPP **(A–F)** or scrambled-ACPP **(G–L)** in ipsi- and contralateral sites. Mice were sacrificed 24 h, 7 days or 8 weeks after KA injection (*n* = 3 independent experiments, two or three animals injected per condition for each experiment). **(M,N)** Enlarged image of the outlined area in panel **(A)** showing TAMRA Gelatinase- **(M)** or Scrambled- **(N)** ACPP fluorescence in the ipsilateral hippocampus. Scale bars: **(J)**, 500 μm, **(N)** 250 μm.

**Figure 5:**
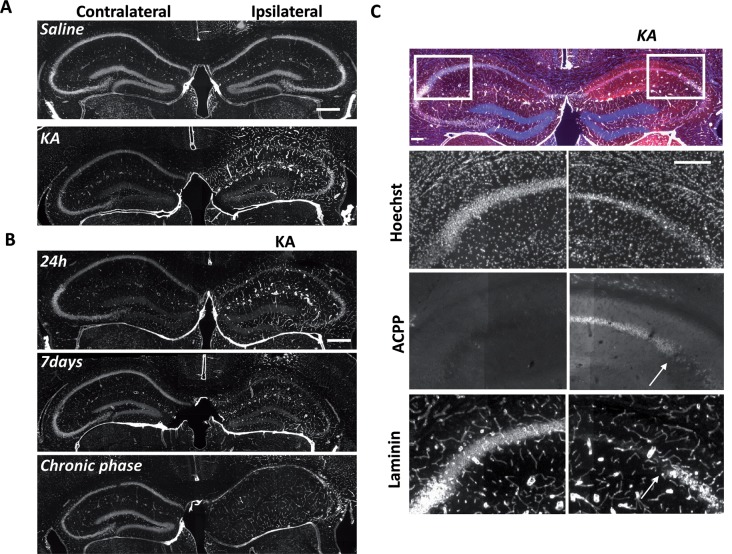
Uptake of ACPP is superimposed to loss of neuronal laminin in KA treated brain.** (A)** Laminin immunoreactivity in saline or KA-injected hippocampus 24 h after injection. **(B)** Progressive changes of laminin expression during the different phases of epileptogenesis. **(C)** Magnification of laminin loss in CA1–CA2 region 7 days after KA injection. The white arrow shows the limit between ACPP uptake and intact laminin (right panels), whereas no TAMRA fluorescence is detectable in the contralateral side and laminin is intact. TAMRA ACPPs (red), Laminin (white), Hoechst (blue). Scale bars: **(A**,**B)** 500 μm, **(C)** 200 μm.

At the site of KA injection, the spatial distribution of TAMRA fluorescence changed over time, revealing specific patterns of gelatinase activation throughout the epileptogenic process (Figure 4). Kinetics in the ipsilateral side is the most informative (Figures 4D–F,M). Twenty-four hours after KA injection, red fluorescent positive cells appeared in CA1, in the hilus, and to a less extent in the granular cell and molecular layers (Figures 3B, 4D,M). These data are as per previous studies showing MMP activation by status epilepticus (Szklarczyk et al., 2002; Jourquin et al., 2003), and therefore they validate the ACPPs. Seven days after KA injection, this fluorescent uptake was reinforced, with stronger staining visible in CA1 and in dispersing granular cell layer (Figures 4E,M). CA1 is known to be the region where strong rearrangements and cell death occur during epilepsy onset. Indeed, in our experiment, a disturbed laminin staining reported KA-induced cell disorganization and cell death process as soon as 24 h (Figure 5A). Laminin is a key component of the ECM and a gelatinase substrate (Chen et al., 2003; Gu et al., 2005). Much more vessels were also visible in the KA-injected hemisphere correlating with the progressive disappearance of intact cells in CA1 and dentate gyrus (Figure 5B; Sarkar and Schmued, 2010). In the KA-treated hippocampus, the increased ACPP uptake appeared as a negative picture of laminin staining (Figure 5C), highlighting gelatinase activity in direct proximity of disorganized cells. In the chronic phase (8 weeks after KA injection, Figures 4F,M), red fluorescence stained cells in the diffuse CA1 and the expanded granular cell layer. In the contralateral side, ACPPs localized in CA1 areas 24 h and 7 days after KA injection and in a sparse punctiform staining in the chronic phase. Vessels in stratum lacunosum moleculare were also stained.

Differences between saline (Figures 4A–C,M) and KA injections (Figures 4D–F,M) were detected. In saline-injected mice, contralateral sites exhibit almost no TAMRA fluorescence. In contrary, ipsilateral injection shows a uniform sparse punctiform staining in the whole hippocampus. This staining, which may reflect a basal gelatinase activation due to inflammation caused by the injection itself, is more important after 1 week of exposure but negligible compared to the KA-injected hippocampus (magnifications of the ipsilateral side are provided in Figure 4M). No particular staining was noticed with saline injections during the chronic phase, except in vessels.

Finally, the cleavage-resistant scrambled peptide was used as control (Figures 4G–L,N) and indicates TAMRA fluorescence background. In the saline-injected hippocampus (Figures 4G–I), no TAMRA fluorescence uptake was detected in the contralateral side and slight staining in microvasculature was noticed in the ipsilateral side (Figure 4N). In the KA-injected hippocampus (Figures 4J–L,N), a weak red signal probably due to neuronal death was observed in CA1 structure in the ipsilateral side only, but much weaker than the fluorescence of gelatinase ACPP samples. The scrambled peptide, therefore, validated the specificity of fluorescence uptake, essentially induced by gelatinase substrate cleavage.

### ACPPs Reveal Cell-Type-Specific Kinetics of Gelatinase Activity During Epileptogenesis

**Figure 6:**
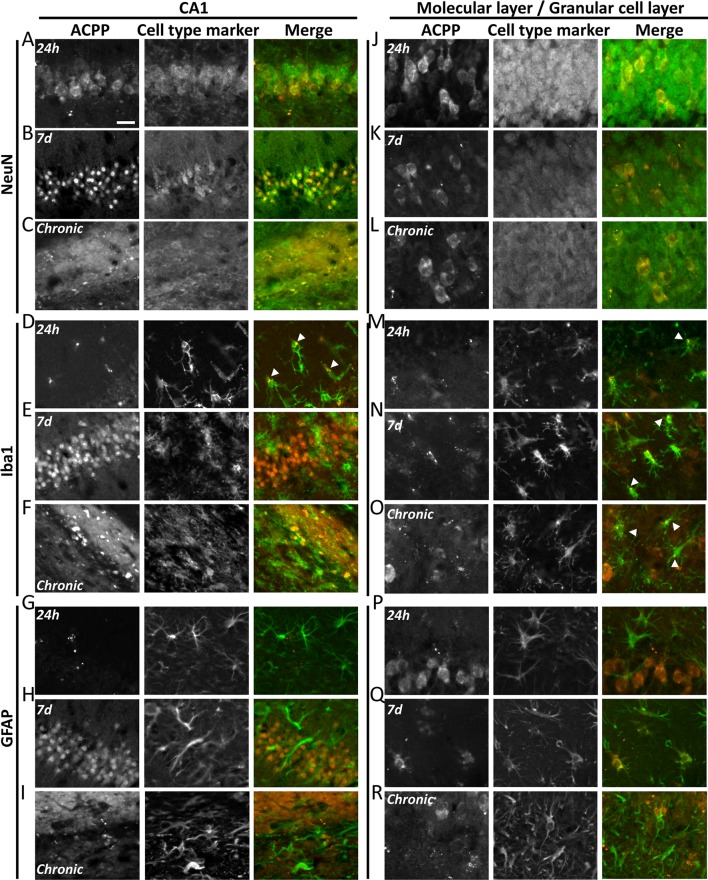
ACPPs reveal cell-type-specific kinetics of gelatinase activity during epileptogenesis. The gelatinase spatiotemporal activation profile was observed with gelatinase-ACPP uptake in CA1 and molecular and granular layers of KA-injected hippocampi at 24 h, 7 days after KA injection or during the chronic phase. Slices were stained with neurons marker NeuN **(A–C,J–L**), microglial marker Iba1 **(D–F,M–O**) or astrocytic marker GFAP **(G–I,P–R**). Merge of the cell type marker (green) and TAMRA ACPP (orange) are shown. *n* = 3 independent experiments, two or three animals injected per condition for each experiment, three slices per mouse were used. Scale bar 20 μm.

To investigate in which cell type gelatinases are activated, we performed immunostaining with markers for neurons (NeuN), microglia (Iba1) and astrocytes (GFAP; Figure 6).

In the KA-injected hippocampus, 24 h after injection gelatinase activity reported by fluorescence uptake correlated with NeuN in CA1 neurons (Figure 6A). A sparse homogenous staining of microglia was detected in the same area (Figure 6D). After 1 week, the fluorescence uptake in neurons in the stratum pyramidale was further increased (Figure 6B), together with neurons from the granular cell layer and hilus (Figure 6K). We noticed a concomitant increase of gelatinase activity in microglia and astrocytes from the molecular layer (Figures 6N,Q). Finally, in the chronic phase, dispersed neurons from the stratum pyramidale and granular cell layer displayed gelatinase activity (Figures 6C,L). At that stage, microglia still displayed gelatinase activity in molecular and granular cell layers, while astrocytes did not (Figures 6O,R). We also noticed in the contralateral part a sparse ACPP positive microglia staining in the chronic phase (data not shown), revealing that inflammation also occurs in the contralateral side of the epileptic brain. In saline-injected conditions the punctiform ACPP fluorescence uptake corresponded to Iba1 positive cells, confirming our previous hypothesis that a basal gelatinase activation by microglia was due to the injection *per se* (not shown).

These data reveal a cell-type-specific temporal and spatial pattern of activation throughout the epileptogenic process. In particular, the common marker along the process is the microglia, confirming an essential role for neuroinflammation in epileptogenesis.

## Discussion

The present work reports the *in situ* gelatinase activation during epileptogenesis using ACPPs. To this end, we engineered a biosensor which enters cells upon gelatinase activation. Cell fluorescence uptake of ACPPs but not uncleavable scrambled forms validate the specificity of the biosensor to report gelatinase activity. This gelatinase activity appears as the negative picture of an intact ECM stained by laminin, confirming that the biosensor is staining cells undergoing remodeling in the vicinity of ECM disruption. Cellular remodeling reported by the ACPPs confirmed the main features of KA-induced epileptogenesis (Pernot et al., 2011), in particular the fact that CA1 is one of the main regions suffering strong rearrangements during epilepsy onset, therefore validating the utility of ACPPs as a biomarker of cellular reorganizations during epileptogenesis. Taking gelatinase-sensitive ACPPs as biomarker of cellular remodeling, we here refine the spatio-temporal pattern of specific cell-types disorganization during epileptogenesis. We found that structural changes in CA1 during status epilepticus, in particular in neurons from the stratum pyramidale in the ipsilateral side, is followed shortly after by dentate gyrus disorganization from hilus to granular cell and molecular layers. In the epileptic brain, cellular remodeling still occurs in diffuse CA1 and expanded granular cell layer. Microglia activation appears as an early phenomenon, lasting all along the epileptogenesis process, while astrocytes remodeling is more transient. Interestingly, these rearrangements are not restricted to the ipsilateral side since in the contralateral hippocampus, ACPPs reports cellular rearrangement in CA1 area 24 h and 7 days after KA injection and a sparse punctiform staining in the chronic phase, due to weaker but prolonged inflammatory processes in the epileptic brain. In KA-injected mice, the absence of fluorescence background with scrambled-ACPPs (no cell death) in the contralateral side together with gelatinase-ACPPs uptake in microglia cells may support a role of neuroinflammation in neuronal cell death protection (Zattoni et al., 2011).

Our study shows that only isolated cells incorporate the ACPP but not surrounding cells, suggesting that the cleaved peptide does not diffuse away from the gelatinase activity but is rather up-taken immediately. We took a profit on this property to identify specific cell-types undergoing remodeling during epileptogenesis. An interesting point is the ACPP colocalization in activated microglial cells in the hippocampal region suggesting a significant role of gelatinases in neuroinflammation as well as the importance of inflammation in epileptogenesis. This observation is consistent with the fact that microglia are among the main sources of gelatinases while they play an important role in epileptogenesis (Kalozoumi et al., 2018). We used gelatinase ACPPs in a preclinically relevant mouse model of TLE, achieved by intrahippocampal application of KA. The initial status epilepticus triggers a massive cellular reorganization over a few days/weeks (early phase), followed by spontaneous chronic seizures around 3 weeks post-SE. This model leads to severe alterations, such as ipsilateral loss of CA1 neurons, dentate gyrus dispersion, and focal hippocampal seizures and inflammation. Those events are spatiotemporally restricted and imply a cell-specific and activity-driven modification of the pericellular environment. In this context, gelatinases are locally secreted at excitatory synapses with proteolytic activity in extracellular space, especially close to dendritic spines of hippocampal neurons following KA stimulation (Konopacki et al., 2007). The localization of ACPPs’ is following previous findings showing an upregulation of MMP-9 expression (mRNA and protein levels) and enzymatic activity in the hippocampal dentate gyrus after KA treatment (Zhang et al., 1998; Szklarczyk et al., 2002; Wilczynski et al., 2008). Yet Szklarczyk et al. (2002) showed stronger activity in molecular and granule cell layers whereas, in our experiments, ACPPs uptake happened more in CA1 and hilus after 24 h of exposition, where cell death occurs. However, these differences in gelatinase activation might be due to nuances in animal models, as previous studies were performed mainly 24 h only after KA injection and KA was administered intraperitoneally. The time course after status epilepticus and the severity of the cellular alterations depend on the studied model and can thus affect the profile of molecular changes. Other epileptic models could be refined using ACPPs. Finally, here we focused on the hippocampus, but some sparse staining was present in other brain areas such as cortex and striatum (data not shown) which could be explained by ACPPs uptake far from the KA application site. An alternative hypothesis is that ACPPs stain hippocampal projections, which would allow reporting with high spatio-temporal resolution neuronal networks undergoing remodeling during epileptogenesis. To get the full picture of cellular remodeling during epileptogenesis, gelatinase activity in other brain regions could also be explored.

Tools are required to assess gelatinase activity *in vivo* and *in vitro* to decipher their various functions such as extracellular remodeling. ACPPs display a polycationic cell-penetrating part composed of D-arginine residues attached *via* a cleavable L-amino acid linker (PLGLAG) to a matching polyanionic sequence of D-glutamate residues. ACPPs adopt a hairpin confirmation due to neutralization between the polyanionic amino acids and the cell-penetrating polycations. When the linker is cleaved by active gelatinases, the two parts dissociate and ACPPs deliver their payload inside the cell (here a TAMRA fluorescent molecule) *via* endocytosis. Our results are consistent with prior findings that ACPPs can be cleaved by secreted MMP-2/-9 and the released CPPs are incorporated into cells displaying the gelatinase activity, for example, tumor cells (Jiang et al., 2004; Aguilera et al., 2009; Olson et al., 2009) and ischemic zones (Chen et al., 2017).

Different techniques to detect MMP-2/-9 proteolytic activity have been used, including the commonly employed *in situ* zymography, based on dye-quenched-gelatin, a fluorogenic gelatinase substrate. The main drawback of these probes is their limited spatiotemporal resolution. A FRET-based biosensor was also recently described (Stawarski et al., 2014) but its use requires exogenous surexpression. In contrast, ACPPs appear as a more reliable and ready-to-use technique to detect *in vivo* MMP-2/-9 activity. The main advantages of ACPPs are the possibility to be used *in vivo*, the fact that they are readily imageable (contrary to the indirect classical zymography technique), and can be adapted to other proteases. We chose to detect the total activation of gelatinases instead of the cleavage of one specific substrate to reliably report ECM disruption and associated cellular remodeling; but on the other hand, in our experiments, we cannot discriminate the involvement of MMP-2 vs. MMP-9, although the latter seems to be the predominant enzyme responsible for these processes (Khomiak and Kaczmarek, 2018). The next improvements would include the design of an MMP-9-specific ACPP. Among the wide range of various substrates for MMP-9, the PRSLS sequence described by Fudala et al. (2012) could be a good candidate. Furthermore, ACPPs could be visualized with the recently developed brain optical clearing technique iDISCO for a 3D mapping of gelatinase activity in the epileptogenic brain at different times. This approach would offer a global overview of proteolytic events, including long-range projections that might be affected by the local KA treatment.

Efforts to improve the precise temporal relationship of cellular reactive changes during epileptogenesis could provide biomarkers (Engel and Pitkänen, 2019) and promote therapeutic intervention in the epileptogenic process (Pitkänen and Lukasiuk, 2011; Goldberg and Coulter, 2014). Because gelatinases are released in a specific time and space-dependent manner, precise information about their kinetics of activation could be used to target anti-epileptogenic drugs in a controlled-delivery manner, fusing a pharmacological compound to ACPPs instead of the fluorescent TAMRA reporter. Gelatinase activity itself could be tuned in cells undergoing remodeling. MMP-9 is indeed the predominant gelatinase involved in epilepsy and has been proposed to be a potential therapeutic target (Yin et al., 2011). Recent studies on human brain surgery tissues showed an upregulation of MMP-9 in epileptogenic hippocampal lesions of patients with TLE (Li et al., 2012; Konopka et al., 2013; Quirico-Santos et al., 2013). The search for novel MMP inhibitors is ongoing, and recently a new molecule, DP-b99, was shown to impair epileptogenesis in animals (Yeghiazaryan et al., 2014), but further investigation is needed to achieve their controlled spatiotemporal delivery. All these works open up new therapeutic opportunities.

To conclude, our approach to detect gelatinase activation enables an *in situ* molecular imaging, providing an overall view of their distribution over a period at the cellular level after status epilepticus and revealed a microglia-neurons joint involvement during epileptogenesis. Thus ACPPs are *in vivo* targeting agents able to investigate the contribution of gelatinases in physio-pathological processes. Their use as molecular imaging probes is an interesting approach for visualizing enzyme activity and may ultimately allow targeting synaptic dysfunction in neurological disorders, such as the pathogenesis of epilepsy.

## Data Availability Statement

All datasets generated for this study are included in the article/Supplementary Material.

## Ethics Statement

The animal study was reviewed and approved by the French Ministry for Agriculture (2010/63/EU, file # 2017011617122099) in accordance with the European Communities Council Directive, and supervised by the IGF institute’s local Animal Welfare Unit (CEEA-LR36).

## Author Contributions

NB, BG, LF, FB, and JP designed the experiments, analyzed and interpreted the data. NB performed all molecular biology and *in vitro* experiments. BG, FB, and JA performed intrahippocampal kainate and ACPPs injections all along the epileptogenic process. NB performed tissue preparation, immunohistochemistry and image acquisitions with the help of BG. NB and JP wrote the manuscript with input from FB and BG. JP supervised the project.

## Conflict of Interest

The authors declare that the research was conducted in the absence of any commercial or financial relationships that could be construed as a potential conflict of interest.

## Footnotes

^1^https://scalablebrainatlas.incf.org

## Supplementary Material

FIGURE S1 Anti-beta-dystroglycan Western Blot of stimulations of endogenous gelatinases in cultured neurons.

FIGURE S2 Anti-GAPDH Western Blot (loading control) of stimulations of endogenous gelatinases in cultured neurons.
