# Supplementary figures and images for: Gelatinase Biosensor Reports Cellular Remodeling During Epileptogenesis
Source: Front Synaptic Neurosci. 2020 Apr 21;12:15. doi: 10.3389/fnsyn.2020.00015 (PMC7186352; doi:10.3389/fnsyn.2020.00015)

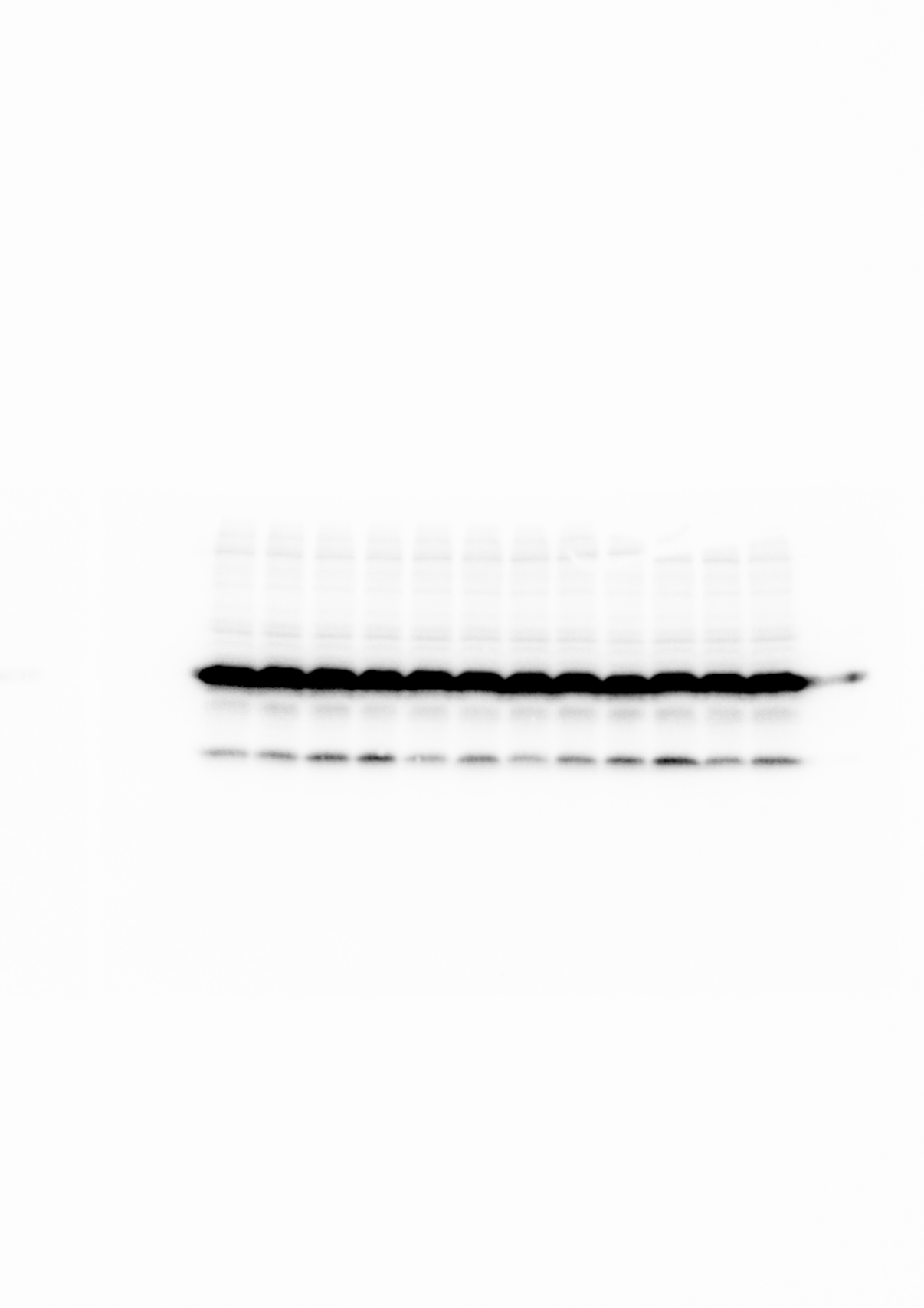

Supplement: FIGURE S1 — Anti-beta-dystroglycan Western Blot of stimulations of endogenous gelatinases in cultured neurons. [file Image_1.TIF]

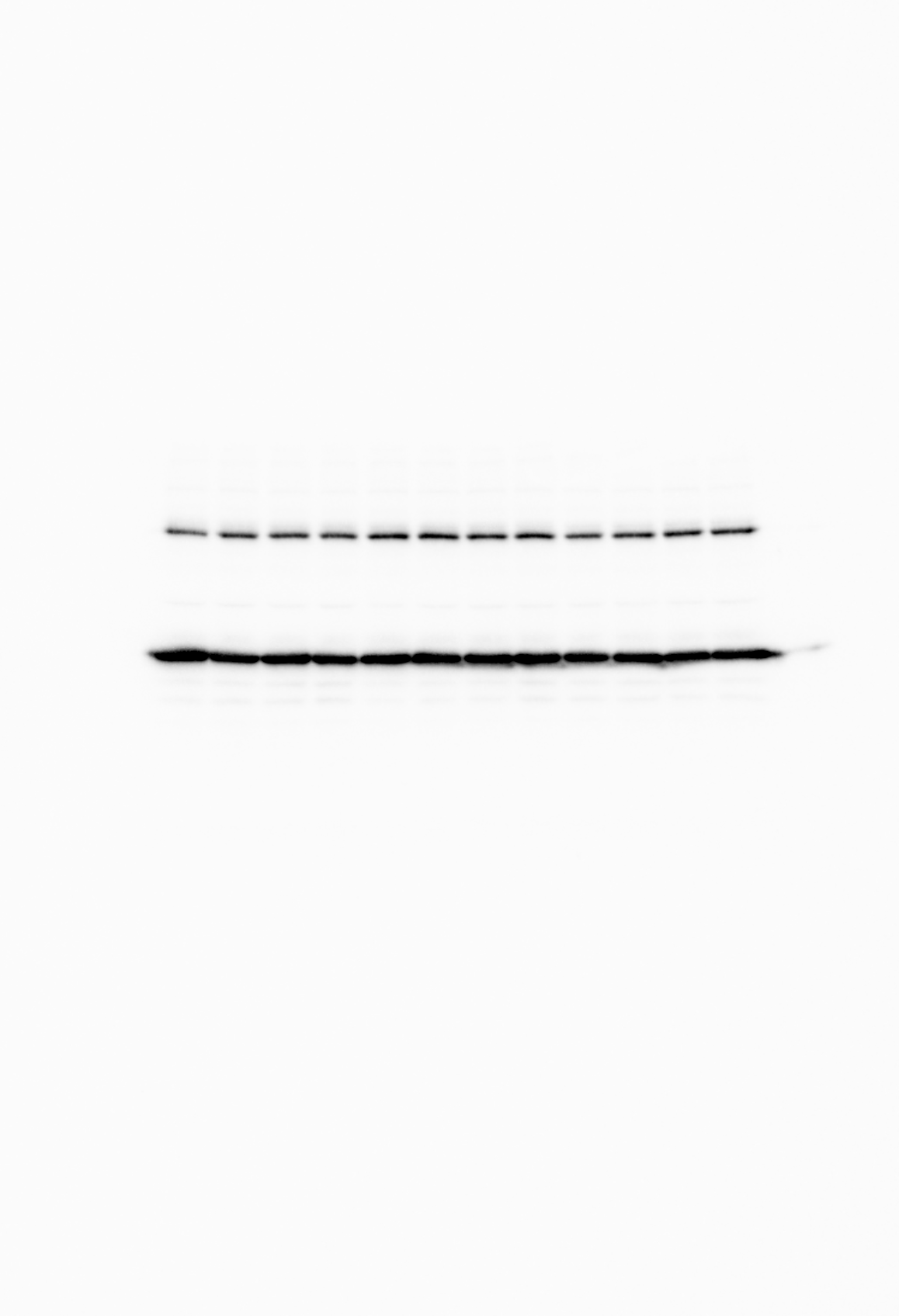

Supplement: FIGURE S2 — Anti-GAPDH Western Blot (loading control) of stimulations of endogenous gelatinases in cultured neurons. [file Image_2.TIF]
